# Interactive Elaborative Storytelling: Engaging Children as Storytellers to Foster Vocabulary

**DOI:** 10.3389/fpsyg.2019.01534

**Published:** 2019-07-05

**Authors:** Enni Vaahtoranta, Jan Lenhart, Sebastian Suggate, Wolfgang Lenhard

**Affiliations:** ^1^Department of Educational Science, University of Regensburg, Regensburg, Germany; ^2^Department of Psychology, University of Würzburg, Würzburg, Germany

**Keywords:** storytelling, shared reading, language intervention, preschool, language development

## Abstract

Positive effects of shared reading for children’s language development are boosted by including instruction of word meanings and by increasing interactivity. The effects of engaging children as storytellers on vocabulary development have been less well studied. We developed an approach termed *Interactive Elaborative Storytelling (IES)*, which employs both word-learning techniques and children’s storytelling in a shared-reading setting. To systematically investigate potential benefits of children as storytellers, we contrasted this approach to two experimental groups, an *Elaborative Storytelling* group employing word-learning techniques but no storytelling by children and a *Read-Aloud* group, excluding any additional techniques. The study was a 3 × 2 pre-posttest randomized design with 126 preschoolers spanning 1 week. Measured outcomes were receptive and expressive target vocabulary, story memory, and children’s behavior during story sessions. All three experimental groups made comparable gains on target words from pre- to posttest and there was no difference between groups in story memory. However, in the Elaborative Storytelling group, children were the least restless. Findings are discussed in terms of their contribution to optimizing shared reading as a method of fostering language.

## Introduction

Substantial discrepancies in language development exist in preschool age, they tend to persist throughout the school years, and rarely resolve (Biemiller and Slonim, 2001). Because children’s language skills at school entry – especially their vocabulary skills – are a powerful predictor of later academic achievement, resolving these discrepancies is a central goal of preschool education. The language environments of children vary greatly: children of parents with a high socio-economic status (SES) roughly hear three times as many words over the course of 1 week than children from low-SES households (Hart and Risley, 1995). In terms of qualitative differences in language exposure, the maternal speech of high SES homes contains more tokens and longer mean lengths of utterance than speech of low SES households (Hoff, 2006) and lower SES mothers use a less varied vocabulary and syntactic structures (Hoff, 2003; Huttenlocher et al., 2010).

Differences also exist in the home language environments (HLEs) that children are exposed to (Burgess et al., 2002; Sénéchal and LeFevre, 2002; Weigel et al., 2006). These differences are reflected in children’s language skills. Biemiller and Slonim (2001) found that second grade children in the highest achieving quartile had an average estimated vocabulary of twice that of children in the lowest quartile. Data from vocabulary test norming projects show that already at the age of five, the most proficient children (i.e., 98th percentile) exhibit a vocabulary knowledge that the poorest two percent of the population need a further 12 years to catch up – at age 17 (Lenhard et al., 2015). Considering the importance of early vocabulary skills for later reading success and in turn, academic success (Cunningham and Stanovich, 1997; Roth et al., 2002; Sénéchal et al., 2006; Suggate et al., 2014), effective interventions are needed to reduce these discrepancies.

Shared reading is an effective way of fostering children’s language abilities and it is widely used by both parents and preschool educators alike (National Early Literacy Panel, 2008). Typically, meta-analyses underline the impact of the amount and type of shared reading on the vocabulary development (Mol et al., 2008; Mol and Bus, 2011; Farrant and Zubrick, 2013). In its simplest form, shared reading consists of reading a story with or to children (e.g., Lenhart et al., 2018), but can be enriched with additional instructional or interactive techniques. Examples of techniques employed include: providing word definitions (Chlapana and Tafa, 2014; Damhuis et al., 2014), initiating interactions on the semantic content (Maynard et al., 2010; Crevecoeur et al., 2014), or involving children in decoding text (Robbins and Ehri, 1994). There is a substantial body of research indicating beneficial effects on vocabulary when such techniques are employed, especially in contrast to an incidental and non-systematic exposure to novel words (Elley, 1989; National Reading Panel, 2000; Penno et al., 2002; Coyne et al., 2007; Marulis and Neuman, 2010; Maynard et al., 2010).

According to the meta-analysis of Marulis and Neuman (2010), approaches focusing directly on teaching vocabulary (so-called *explicit approaches*) are more effective at promoting vocabulary learning than *implicit approaches*, which by definition do not include deliberate teaching of words. Explicit approaches employ different techniques such as definitions, examples, and discussion of words (Penno et al., 2002; Coyne et al., 2007; Nelson and Stage, 2007; Nielsen and Friesen, 2012; Damhuis et al., 2014; McKeown and Beck, 2014). It can be argued that word-teaching techniques fall on a continuum from purely implicit at one end, to purely explicit techniques at the other end. For example, discussing word meanings and providing definitions of words would be close to the explicit end, whereas showing pictures accompanying the story would fall close to the implicit end.

It has been suggested that the explicit teaching of words during storytelling requires interrupting the story, often switching from a narrative story genre to a non-fictional encyclopedic genre, potentially drawing attention away from the story itself or even undermining listening motivation (e.g., Suggate et al., 2014; Vaahtoranta et al., 2018). Accordingly, Vaahtoranta et al. (2018) developed an alternative to explicit approaches called *Elaborative Storytelling*. In this approach, instructional techniques are designed to support vocabulary learning by providing more contextual information and drawing children’s attention to certain words while excluding explicit teaching of words. Examples of techniques include rhetorical questions, elaborations, and providing synonyms. In contrast to explicit approaches, these techniques were intended to be more subtle and not to take children’s attention too far away from the story plot and thus fall somewhere between explicit and implicit approaches. In Vaahtoranta et al. (2018), children heard two stories across 3 days with either strictly explicit word-learning or more elaborative techniques accompanying target words. Measures assessed target-word gains and story-retelling alongside children’s social behavior. Vocabulary gains were generally similar for both approaches, except that explained words were learned better in the explicit conditions, and that children were more attentive in the elaborative condition. Thus, elaborative techniques could present an alternative or addition to explicit approaches in shared reading, with possible benefits for children’s engagement.

A second element that varies in shared reading is the level of interaction with the children, referring to which degree children actively participate in the story activity. Such interactivity can take different forms and focus on either dialogic or narrative aspects. Dialogue, or conversation, is cooperative communication that involves two or more participants (Petersen, 2011). In language interventions, dialogue is often employed by involving children in a discussion about the delivered content or new words, the most prominent example being *dialogic reading* (Whitehurst et al., 1994). In dialogic reading, parents and educators endeavor to involve children more actively in shared reading by encouraging them to talk about the story, asking open questions, and discussing word meanings or content (Mol et al., 2008). Single studies indicate positive effects of dialogic reading on vocabulary development (Chow et al., 2008; McBride-Chang, 2012). A meta-analysis (Mol et al., 2008) found moderate effects for general vocabulary (*d* = 0.42) and large effects for expressive vocabulary (*d* = 0.59). However, at-risk children benefited less than their peers and kindergarten children profited less than preschool children. A possible reason is that the requests implemented in dialogic reading, such as to make inferences, are too demanding for children with low language skills (Mol et al., 2008).

Beneficial effects of the active involvement of children can also be found in less formal settings. For example, Reese et al. (2010) found that children benefitted from elaborative reminiscing, in which parents encourage children to expand on their talk about past events. Mothers were trained to talk about past events in a detailed way and to ask children open-ended questions. The children, whose mothers were trained in elaborative reminiscing, showed enhanced autobiographical memory and narrative compared to the control group.

In contrast to dialogic styles, narration or storytelling is a form of discourse in which one person imparts content while others listen (Petersen, 2011). As such, storytelling is a “sophisticated decontextualized form of oral language” (Spencer et al., 2014, p. 244), places a great demand on the narrator’s expressive language, and requires him/her to use complex language (Petersen, 2011). Thus, interventions training children in storytelling could present an avenue for promoting children’s language skills, especially their expressive language. Indeed, approaches employing narration by children indicate positive effects on language skills, shown both in individual (Swanson et al., 2005; Spencer et al., 2014) and group settings (Nicolopoulou et al., 2015; Spencer et al., 2015; Licandro, 2016).

To date, studies have mostly focused on narrative skills as the main outcome variable, showing that children’s narrative skills can be fostered by practicing retelling, acting out, or inventing stories. In a narrative peer intervention (Licandro, 2016), Turkish-German bilingual children with low and children with high narrative skills were trained to tell each other stories with the aid of pictures over a course of 10 weeks. Children who took part in the peer-intervention improved their narrative skills significantly more than the two control groups. However, we were only able to identify one study that investigated word-learning from retelling. In Nielsen and Friesen (2012), children participated in a 12-week storybook intervention with vocabulary teaching and retelling. Here, the intervention group made more gains in storybook vocabulary than the no-treatment control group. The authors also report that the intervention group made more gains on a standardized measure of semantics than the control group, however, without sufficiently reporting statistical parameters needed for substantiating the difference of gain scores, and based on a rather low group size of *n* = 14. Consequently, more work is needed to replicate and extend these findings.

## Current Study

Both the active involvement of children in shared-reading situations and the employment of explicit word-learning techniques have been shown to be beneficial for language learning (Mol et al., 2008; Marulis and Neuman, 2010). Elaborative techniques present an alternative to explicit word-learning techniques, with potential benefits for children’s attention for the story (Vaahtoranta et al., 2018). Regarding interactivity, findings are mixed – although positive effects of dialogic reading have been found for children with normal and high language skills, these do not necessarily transfer to children with low language skills (Mol et al., 2008). Retelling stories could present an alternative way to foster expressive language, via encouraging learners to implement new words and structures into their language repertoires. Positive effects of retelling have been shown for narrative skills (Nielsen and Friesen, 2012; Spencer et al., 2014; Nicolopoulou et al., 2015; Licandro, 2016); however, effects on vocabulary have been largely ignored.

Building on these findings, we sought to increase the interactivity of current approaches by incorporating elements of elaborative storytelling (Vaahtoranta et al., 2018) coupled with the potential benefits of using children as additional and active storytellers. Accordingly, we compared three experimental groups: *Interactive Elaborative Storytelling (IES)*, *Elaborative Storytelling*, and *Read-Aloud*. *IES* includes (a) the elaborative instructional techniques from Vaahtoranta et al. (2018) for fostering vocabulary learning and (b) retelling of the stories by the children, similarly to Nielsen and Friesen (2012), as an additional tool to promote expressive language. Instructional techniques include rhetoric questions, comments, paraphrasing, and synonyms, and are designed to help word learning by drawing attention to certain words and by giving more contextual information, at the same time engaging children in the story and thus helping to keep their attention (Vaahtoranta et al., 2018). Similarly to Nielsen and Friesen (2012), children were encouraged to increasingly retell the stories across the storybook sessions and were supported with illustrations of the stories. The instructor receded into a supporting role, giving prompts when needed and supporting learning of new words. This approach thus employs a form of scaffolding, which is also inherent in dialogic reading (Walsh and Hodge, 2016).

To tease apart effects of retelling from effects of elaborative instructional techniques, *Elaborative Storytelling* was added as an experimental group. The *Elaborative Storytelling* group included elaborative word-learning techniques from the Vaahtoranta et al. (2018) study but not retelling by the children. The *Read-Aloud* group and was further introduced to control for effects of simple shared reading and general intervention effects and hence excluded any instructional techniques. These three experimental groups were compared in a randomized and controlled small-group study in preschool/kindergarten spanning 1 week. In order to measure both shallow word recognition and deeper word processing (Blewitt et al., 2009; Lenhart et al., 2019), word learning was measured with a receptive and an expressive vocabulary task. A measure of story memory was furthermore added, to capture not only word learning but also story comprehension (Walsh and Hodge, 2016).

Although we expected gains in story vocabulary in all three conditions, we assumed gains to be highest in the IES condition. This effect was expected to be higher for expressive target words compared to receptive target words due to the expressive nature of the intervention. Furthermore, we expected story memory to be higher for the IES condition than the other conditions. Finally, we explored effects on children’s behavior, more specifically how restless and how interested children appeared during story sessions. Here, we were not certain in which directions effects would manifest. On the one hand, the added interactivity in the IES condition could boost children’s interest and engagement in the stories and hence lead to less restlessness. On the other hand, this kind of interactivity in a group of children could also possibly lead to more disturbance in story sessions and requires effective moderation by the experimenters. It is also conceivable that the Elaborative Storytelling group would be least restless because it includes prompts designed to engage children more in the story but has less interactivity than the IES group.

## Materials and Methods

### Participants

The first two authors recruited preschools directly by contacting them via telephone. Five kindergartens in two cities in the south of Germany took part in the study. In total, the parents of 140 children, 61 in the first and 79 in the second city, agreed to participate by providing informed, written consent. Three children were missing at pre- and posttest and 11 children participated in only one story session or in zero sessions and were excluded from analyses, resulting in a final sample of 126 children. Fifty percent of the children were female and mean age was 5.0 years (*SD* = 7.5 months), ranging from 4.0 to 6.7 years. A parent demographics questionnaire showed that 33% of mothers and 30% of fathers had obtained a university degree, and that 32% of the parents were born outside of Germany. The parents in the sample were thus more highly educated than the general German population (17%) and the proportion of parents born outside of Germany was higher than in the general population (18%) (Statistisches Bundesamt, 2018). For 64% of the families, the language spoken at home was German, for 35% another language or both German and another language. The project was approved by the university human ethics committee.

### Design and Procedure

**FIGURE 1:**
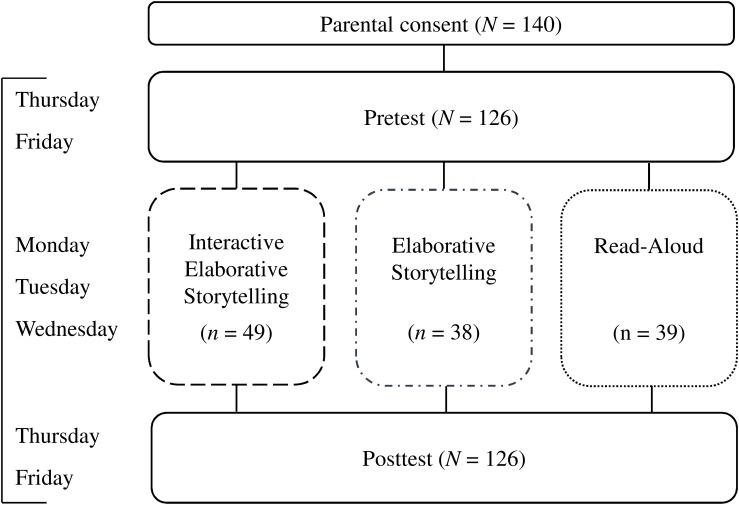
Overview of the experimental design and procedure.

The design was a mixed, repeated-measures 3 × 2 experimental design with the experimental group as between-subjects factor (IES vs. Elaborative Storytelling vs. Read-Aloud) and time as within-subjects variable (pre- vs. posttest). The experiment spanned one and a half weeks per kindergarten, with pretest on Thursday and Friday, story sessions on Monday, Tuesday, and Wednesday, and posttest again on Thursday and Friday. See Figure 1 for an overview of the design. Children were randomly assigned to the experimental groups in a pull-out fashion. There were a total of 22 groups, seven groups in the Read-Aloud and the Elaborative Storytelling conditions and eight groups in the IES condition, with four to seven children in each individual group. A total of 10 trained student research assistants conducted both testing and experimental groups. In each storytelling session, two experimenters were present, one as storyteller and one as observer, in alternating roles. Pre- and posttest each lasted approximately 30–45 min per child.

### Measures

All measures and materials were in German.

#### Selection of Target Words

Target words were selected from the Vaahtoranta et al. (2018) study, in which the same two stories were employed. From the original 40 target words, 20 words, 10 per story, were chosen. We applied the same criteria as in Vaahtoranta et al. (2018) for selecting target words: We ensured that target words (a) had a low frequency of occurrence in everyday language and in children’s books, (b) did not appear more than once in the story, (c) did not appear in the other story used in the study, (d) came without accompanying definitional explanation in the story, and (e) appeared evenly throughout the stories. The selected target words had a maximum absolute type frequency of 11 in the lexical database of children’s literature childLex (Schroeder et al., 2015), indicating that the words were most likely not familiar to the children. We deemed this important to ensure that children had the chance to learn the target words though the story sessions. Examples of target words were are *flink* (“swift”), *glimmern* (“to glisten”), and *Pforte* (a rarely used word for “gate”). To ensure that mere testing did not affect word learning, 10 control words that did not appear in the stories were included in the receptive vocabulary task. The control words were matched to target words on word class (nouns, adjectives, and verbs) and frequency in childLex (Schroeder et al., 2015), *t*(28) = −1.34, *p* = 0.13.

#### Receptive Target Vocabulary

Children’s pretest and posttest receptive target and control word knowledge were measured using an author-generated picture vocabulary test. Children were shown four pictures containing the target picture and three distractors, and were asked to point to the picture corresponding to the test item orally presented by the experimenter. Each picture appeared four times, once as target and three times as a distractor. Scores could range between 0 and 20. The internal consistency of the vocabulary test was estimated as α = 0.66 for posttest. A repeated-measures ANOVA showed that there was no increase in control word knowledge from pretest to posttest, *F*(1,114) = 2.61, *p* = 0.11, and no interaction of time and experimental group, *F*(2,114) = 1.57, *p* = 0.21, indicating that the mere testing of words did not lead to word learning.

#### Expressive Target Vocabulary

To measure deeper word processing (Blewitt et al., 2009; Lenhart et al., 2019), children were also tested for expressive target word knowledge at pre- and posttest using an author-generated test modeled from Coyne et al. (2007) and Loftus et al. (2010). Children were asked to define the words by asking “Can you tell me what *to scold* means?.” If they did not know the answer to the first question, the experimenter asked the follow-up question “Does anything come to mind?” or “Do you maybe have some idea what it could mean?.” To keep children engaged and avoid frustration with difficult words, target items were interrupted by easy items such as *tomato* or *to swim*. Responses to target words were graded independently by two raters according to the following criteria: (a) three points for either a complete explanation or a synonym corresponding to the target word, (b) two points for an approximate explanation or a synonym that does not entirely represent the word’s meaning, (c) one point for a response that showed a distant association with the target word, and (d) zero points for a missing or incorrect answer. Thus, total scores for the 20 target words could range between 0 and 60. Interrater reliability, measured with two-way random single measure intraclass correlations, was good at posttest, ICC(2,1) = 0.88 (Koo and Li, 2016). Internal consistency was α = 0.68 for posttest.

#### Story Memory

Children’s story memory of the presented stories was assessed at posttest with an author-generated task. This task contained five story memory questions per story, 10 in total, for example, “Why was the girl sad?” or “What tasks did the brothers have to perform?,” ranging from simple recalling to questions requiring understanding of the story. Answers were coded independently by two raters according to a scoring plan generated by the first and second author. Children could score zero, one, or two points per question, with a maximum total score of 20. Interrater reliability was excellent, ICC(2,1) = 0.99. Internal consistency was α = 0.73 for both stories.

#### General Vocabulary

In order to check for equivalence of the experimental groups, children were assessed for their general receptive vocabulary using the German adaptation of the PPVT-IV (Lenhard et al., 2015). Here, children are presented sets of four pictures and are asked to indicate the picture corresponding to the word spoken by the experimenter. Items increase in difficulty and the test continues until eight or more errors in a set of 12 are made or the entire test is completed. The PPVT-IV exhibits excellent internal consistency (Cronbach’s α = 0.97; Lenhard et al., 2015).

#### Phonological Working Memory

As an additional measure of equivalence between groups, the subtest *Phonological Working Memory for Non-Words* from the language-assessment battery *Speech Development Test for Three- to Five-Year-Old Children* (SETK 3-5; Grimm et al., 2010) was used to measure children’s phonological working memory performance. This test consists of 18 German-like non-words of increasing length. Items are presented orally, and the child’s task is to repeat them. Internal consistency for the raw score was acceptable (Cronbach’s α = 0.77).

#### Rating of Children’s Behavior

Children’s behavior during the story sessions was rated by an observer immediately after the sessions for how restlessness and how attentive they appeared on five-point Likert scales. Restlessness was defined as disruptive behavior by the children, such as talking about other things during the storytelling, or motor restlessness. Attentiveness was defined as whether children seemed to follow the story and the activities. In 25 out of the total of 66 (38%) of the story sessions, a second observer was present and for these sessions, interrater reliability was calculated resulting in ICC(1,1) = 0.78 for restlessness and ICC(1,1) = 0.37 for interest across all sessions. Due to the low interrater reliability, interest was excluded from the analysis.

#### Parent Demographic Questionnaire

Parents filled out a questionnaire assessing demographic and other background variables. Variables included the country of birth for both parents and the child, the parents’ highest level of education (ranging from 1 = no secondary school qualification to 5 = university degree) and languages spoken at home.

### Materials

#### Stories

The same two stories as in Vaahtoranta et al. (2018), *The Old Woman in the Forest* and *The Queen Bee* from Grimm’s Fairy Tales (Grimm et al., 2003), were used. These stories were deemed to be unfamiliar to the children as well as age-appropriate in content, style, and length. The stories were 778 and 730 words long, and when read aloud, both lasted between 5 and 6 min. The readability indexes (Björnsson, 1968) were calculated using an online tool (Lenhard and Lenhard, 2011) and indicated suitability for children (40 vs. 41). For each story, 10 pictures illustrating the story line were drawn by student assistants to increase children’s attention and support storytelling by the children. The pictures were printed individually and shown to the children throughout the story at preassigned positions.

### Experimental Groups

To systematically examine the effectiveness of different interventional techniques, we included three experimental groups, namely, *Elaborative Storytelling*, IES, and *Read-aloud*. Children were randomly assigned to the groups in a pull-out fashion and attended story sessions on three consecutive days. In each experimental group, the same two stories were covered across the three storytelling sessions. In total, children heard both stories three times. Sessions included a short movement activity before each story in order to help keep children focused.

#### Interactive Elaborative Storytelling

In the IES condition, target words were accompanied with the word learning techniques from the Vaahtoranta et al. (2018) study and children were furthermore included as storytellers. In the first session, the stories were read aloud, with all target words accompanied by word-learning techniques. These techniques were designed to draw children’s attention to what is semantically occurring around the target words and to provide additional contextual information while maintaining the flow of the story. Techniques included paraphrasing and the use of synonyms (e.g., “Can you imagine how that was? It was so exhausting!”; target word: *arduous*), closer descriptions (e.g., “The rings sparkled and glittered like stars.”; target word: *to glisten*), rhetorical questions (e.g., “I wonder, how are they going to accomplish that?”), and questions supporting children’s imagination (e.g., “What do you think, was it a big tree?”) (Vaahtoranta et al., 2018).

In the following sessions, interactivity was progressively increased. In the second session, the experimenter read aloud a paragraph of the story and then asked the children to continue the story. If the children did not know how the story continued, the experimenter read another paragraph and asked again. This continued until the end of the story. In the third session, children were encouraged to tell the whole stories themselves with only little help from the experimenter. Experimenters were trained to react to different situations that could occur in the second and third session. Regarding target words, experimenters encouraged the children to use them in their retellings, for example, if a child used a synonym of the target word or if the word was not used at all. Experimenters were also instructed to encourage all children to participate in storytelling and to avoid the same children always telling the story. For example, if one child was mainly telling the story, experimenters were instructed to say something in the line of “Thank you for telling the story so well. Would someone else like to continue with the story?” and if needed, to encourage quiet or shy children if they would like to continue. Furthermore, experimenters made sure that the full story was told and to complement the storytelling if needed. Possible scenarios and responses were summarized in a flowchart which was given to the students as a guideline in order to make the groups as standardized as possible.

#### Elaborative Storytelling

In the *Elaborative Storytelling* condition, the same instructional techniques as in the IES group were employed, excluding retelling by the children. Elaborations of target words were distributed evenly across the sessions, with four elaborated words in the first and three in the second and third session.

#### Read-Aloud

In order to control for a general intervention effect and the exposure to stories, a Read-Aloud group was included. Here, the stories were read aloud without word-learning techniques and without children taking part in the storytelling. To make this condition comparable to the other groups in both duration and interactivity, each session included one or two tasks from an intervention program for phonological awareness (Küspert and Schneider, 2010). Games included, for example, segmentation of words into syllables or rhyming, lasting approximately 5 min, and were administered after both stories were read aloud.

### Experimenter Training

Experimenters were education students who were trained in conducting the story sessions. Experimenters were trained to read the stories in all conditions in a natural yet exciting way to keep children interested and attentive. This entailed reading at an appropriate pace, employing voice modulation, and frequently making eye-contact with the children. Special attention was paid to target words, making sure that were pronounced clearly and comprehensibly. Experimenters were further trained for the specific requirements of the different experimental conditions. To achieve as much standardization of the intervention sessions as possible, the stories used in the sessions included the word-learning techniques as well as prompts when to show the pictures. This was done to ensure that all word-learning techniques appeared at the right time and that the pictures were shown for a comparable time. Experimenters were furthermore given a flowchart showing appropriate reactions to children’s replies and storytelling in the IES group. For the read-aloud group, experimenters were given specific activities for each session.

### Data Analysis

Analyses were conducted using IBM SPSS 24, and the package *lme4*, version 1.1-14 (Bates et al., 2015) of the software R (version 3.4.4; R Core Team, 2018). The significance of individual regression coefficients was assessed using the package *lmertest* (Kuznetsova et al., 2017). This statistical analysis has advantages over traditional analyses of variance, such as the ability to handle missing data. The relationship between experimental groups and performance on the vocabulary measures at pre- and posttest was investigated with mixed linear regression models. In all analyses, we used dummy coding with the read-aloud group and the pretest serving as reference categories, identified by the value 0. Consequently, the intercept represents the values of the read-aloud group at pretest. The regression coefficients reflect simple effects or simple interaction effects. Performance on the story memory task was analyzed with a simpler linear model without random effects because each subject produced only one measurement for posttest.

### Power Analysis

We conducted a power analysis with the program G^*^Power (version 3.1.9.2; Faul et al., 2009). Because there is no generally accepted method to conduct power analyses for complex linear mixed-effects models to date, we chose to conduct power analyses for the corresponding ANOVA design (2 × 3, within-between-design). Regarding effect size, a conservative estimate for the within-between interaction would be a small- to medium-sized effect (Richard et al., 2003). Given an effect size of *f* = 0.15 (corresponding to Cohen’s *d* = 0.3), power = 0.80, and alpha = 0.05, the total required sample size would be *N* = 111. Thus, given our sample size of 126, we would be able to detect a small- to medium-sized effect.

## Results

### Missing Data

A small proportion (3%) of the language measures (target word acquisition, story memory, or general vocabulary) was missing due to children being sick or otherwise not present at the testing days. Analyses were conducted with listwise deletion. Descriptive statistics for the language variables as a function of group assignment to the three experimental conditions were calculated and these appear in Table 1.

**TABLE 1:** Descriptive statistics for the three experimental groups.

	**Experimental group**											
	**Interactive Elaborative Storytelling (*n* = 49)**				**Elaborative Storytelling (*n* = 38)**				**Read-aloud (*n* = 39)**			
	***M***	***SD***	**Min.**	**Max.**	***M***	***SD***	**Min.**	**Max.**	***M***	***SD***	**Min.**	**Max.**
Age (years; months)	4;11	0;7	4;1	6;1	5;0	0;8	4;0	6;2	5;1	0;8	4;1	6;7
General receptive vocabulary (PPVT-IV)	49.00	9.00	27	64	50.79	10.84	27	71	43.54	8.98	27	67
**Receptive target vocabulary**												
Pretest	5.96	2.19	2	12	6.51	2.79	3	13	5.45	2.02	2	9
Posttest	8.04	3.64	1	15	9.00	3.67	3	17	7.03	2.70	2	14
**Control receptive vocabulary**												
Pretest	2.93	1.34	1	6	3.03	1.30	1	6	2.76	1.63	0	7
Posttest	3.61	1.80	0	8	3.11	1.92	0	7	2.92	1.61	0	7
**Expressive target vocabulary**												
Pretest	1.70	2.17	0	8	1.76	2.15	0	8	0.92	1.68	0	8
Posttest	3.39	3.97	0	17	3.95	4.98	0	18	1.62	2.35	0	9
Story memory	7.53	3.93	0	16	8.72	4.44	0	15	6.90	4.93	0	15

### Equivalence of Experimental Conditions

A MANOVA showed that experimental groups did not differ in participant age, *p* = 0.470, pretest receptive target vocabulary, *p* = 0.136, phonological working memory (standard score), *p* = 0.256, or attendance of story sessions, *p* = 0.817. A significant difference between groups was, however, found on general receptive vocabulary, *p* = 0.001, η_p_^2^ = 0.11, with the read-aloud group displaying a significantly lower standard score on the PPVT-IV (*M* = 43.54, *SD* = 8.98) than the IES group (*M* = 49.00, *SD* = 9.00) and the Elaborative Storytelling group (*M* = 50.79, *SD* = 10.84). Therefore, we included PPVT-IV standard scores as covariates in all analyses. Groups did not differ on language spoken at home (German vs. other language and German), χ^2^(2, *N* = 124) = 0.83, *p* = 0.659, sex, χ^2^(2, *N* = 126) = 2.26, *p* = 0.324, or highest maternal education, χ^2^(2, *N* = 121) = 14.89, *p* = 0.061.

### Receptive Target Vocabulary

Receptive target vocabulary was investigated as a function of experimental group and time, and their interaction, with group and time as fixed effects and subject as random effect, using the function *lmer*. Because groups differed for general vocabulary, the PPVT raw score was added as a covariate [formula: receptive target vocabulary ∼ Time + Group + Group^*^Time + PPVT + (random|subject)]. The regression coefficients of the intervention groups represent difference values between the groups and the read-aloud group at the pretest, while the regression coefficients of the interaction between time and intervention groups reflect differential learning gains between those groups and the read-aloud group.

**FIGURE 2:**
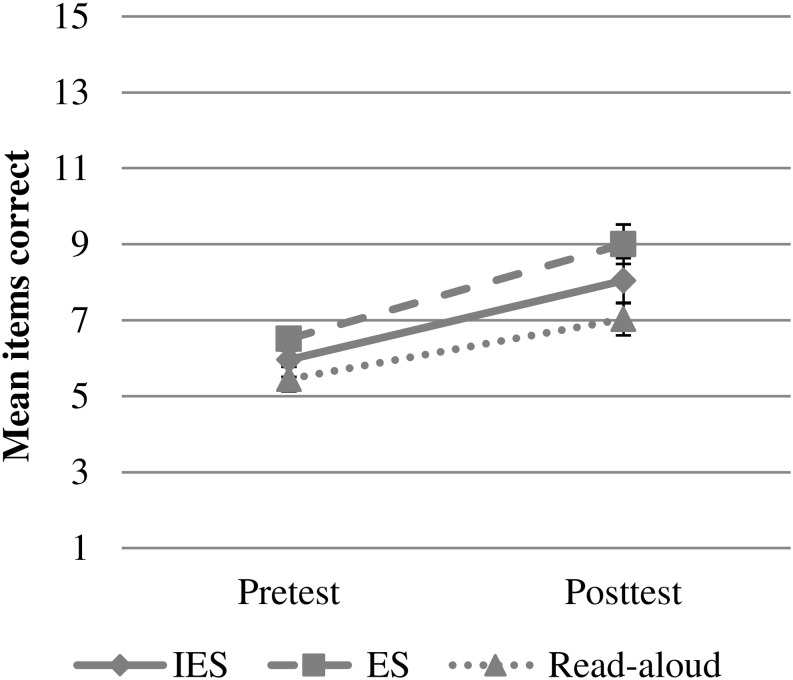
Mean items correct on receptive target vocabulary task at pre- and posttest as a function of experimental group (IES = Interactive Elaborative Storytelling; ES = Elaborative Storytelling). Error bars represent ± 1 standard error.

Results revealed that in the Read-Aloud group, scores on receptive target vocabulary were significantly higher at posttest than at pretest. There was no difference between IES and Elaborative Storytelling (*Estimate* = 0.25, *SE* = 0.58, *df* = 232.45, *t* = 0.42, *p* = 0.673), and the two groups did not differ from Read-Aloud. No differences between groups or interactions were found, indicating that the groups did not differ in increase at pre- or posttest as well as regarding learning gains (see Figure 2). PPVT emerged as significant predictor, thus children with higher scores on PPVT scored higher on the receptive target word task. See Table 2 for a detailed overview of the analyses.

**TABLE 2:** Mixed regression analyses of language outcomes as function of intervention group, time, and general vocabulary (PPVT).

	**Receptive target vocabulary**					**Expressive target vocabulary**			
	***Estimate***	***SE***	***df***	***t***	***p***	***Estimate***	***SE***	***z***	***P***
**Fixed parts**									
(Intercept)	**5.87**	0.43	231.20	13.62	**<0.001**	−**0.53**	0.26	−2.04	**0.041**
Time (posttest)^a^	**1.50**	0.57	122.70	2.64	**0.009**	**0.55**	0.21	2.61	**0.009**
Group (ES)^b^	0.23	0.62	230.76	0.37	0.708	0.26	0.34	0.75	0.454
Group (IES)^b^	−0.01	0.58	231.74	−0.03	0.980	0.59	0.32	1.84	0.065
PPVT sum score^c^	**1.32**	0.19	127.06	7.02	**<0.001**	**0.68**	0.12	5.72	**<0.001**
Time (posttest) × group (ES)	1.04	0.81	122.77	1.28	0.202	0.26	0.26	1.00	0.32
Time (posttest) × group (IES)	0.60	0.76	122.24	0.79	0.434	0.08	0.25	0.31	0.76
**Random parts**									
Intercept variance	0.92					0.99			
Observations	243					247			
N_subjects_	126					125			

*The models were fitted with the lmer-function (receptive target vocabulary) and the glmer-function (expressive target vocabulary) of the *lme4* package. Each model comprised a random intercept for subject as well as time and group as fixed effects. The factor Time was dummy coded (word recognition and word definition: pretest = 0), as was the factor Group (control group = 0). The intercept represents the score of the control group at pretest. The effect of time represents the change in the control group from pretest to posttest. ^*a*^Reference = pretest; ^*b*^reference group = control group; ^*c*^centered. Bold face indicates significant estimates (*p* < 0.05).*

### Expressive Target Vocabulary

Expressive target vocabulary was investigated with intervention group, time, and their interaction as fixed effects and a random intercept for subject. The PPVT raw score was again added as a covariate to the model [formula: expressive target vocabulary ∼ Time + Group + Group^*^Time + PPVT + (random| subject)]. Due to a zero-inflated distribution, the model was fitted to a Poisson distribution using the function *glmer*. As above, the regression coefficients of the intervention groups represent difference values between the groups and the read-aloud group at the pretest, while the regression coefficients of the interaction between time and intervention groups reflect differential learning gains between those groups and the read-aloud group.

**FIGURE 3:**
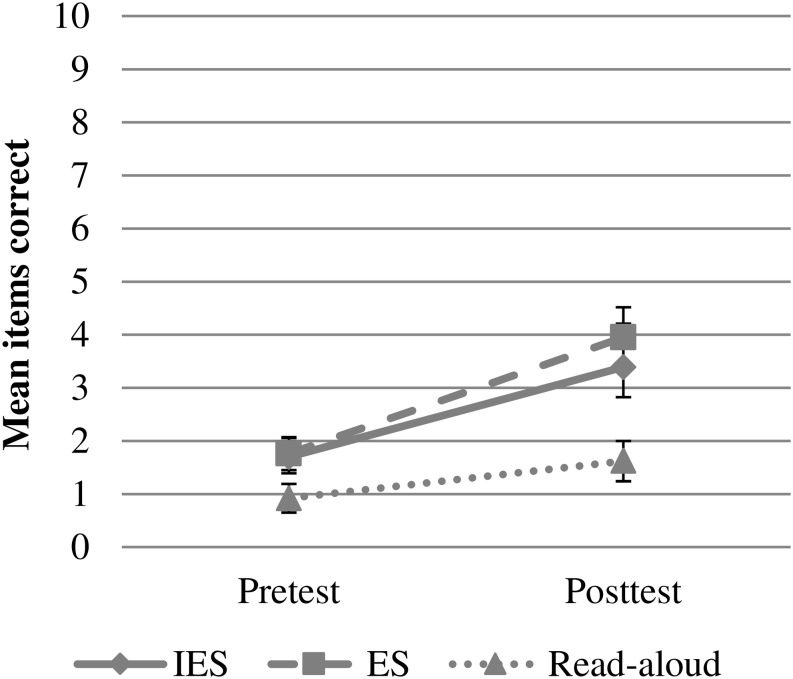
Mean items correct on expressive target vocabulary task at pre- and posttest as a function of experimental group (IES = Interactive Elaborative Storytelling; ES = Elaborative Storytelling). Error bars represent ± 1 standard error.

Results show that for the control group, scores on expressive target vocabulary were significantly higher at posttest than at pretest. Regarding the effect of group, IES scored marginally significantly higher than the Read-Aloud group. IES did not differ significantly from Elaborative Storytelling (*Estimate* = −0.33, *SE* = 0.29, *z* = −1.14, *p* = 0.256). No interactions were found between time and group, meaning that the increase from pre- to posttest did not differ between the groups (see Figure 3). The significant effect of PPVT shows that higher scores on PPVT were related to higher scores on the expressive target vocabulary task. See Table 2 for a detailed overview of the analyses.

### Story Memory

**FIGURE 4:**
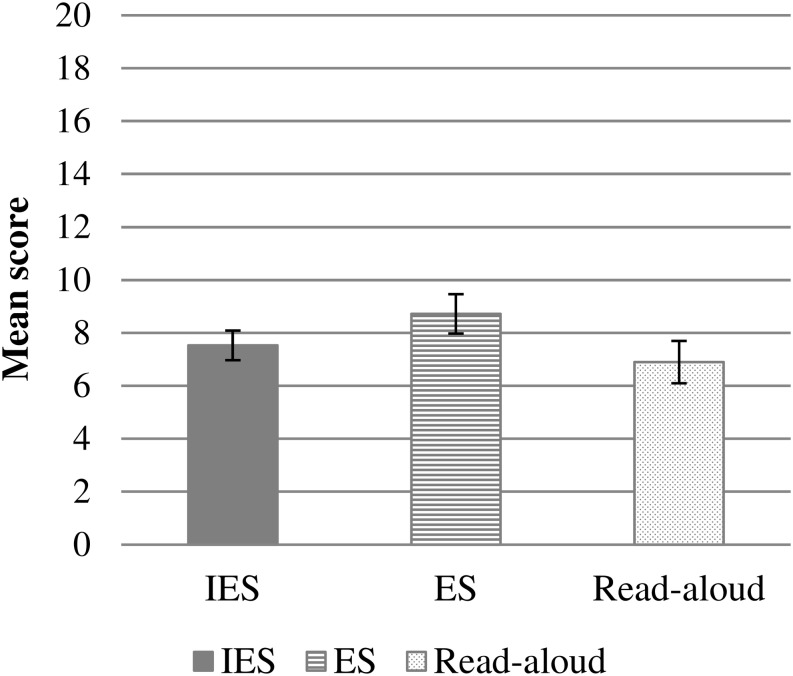
Mean score on the story memory task at posttest as a function of experimental group (IES = Interactive Elaborative Storytelling; ES = Elaborative Storytelling). Error bars represent ± 1 standard error.

Performance on the story memory task was analyzed with a simpler linear model without random effects because each subject produced only one measurement for posttest. Story memory was analyzed with a linear model using the function lm, with group as fixed effect and PPVT as covariate (formula: Story_memory ∼ Group + PPVT). Story memory was only assessed at posttest, and thus time was not included as predictor in this model. The analysis showed no group differences in story memory (see Figure 4) and a strong association of PPVT and story memory, with higher scores on PPVT predicting higher scores on story memory. See Table 3 for the detailed model.

**TABLE 3:** Linear regression analysis of story memory as a function of intervention group and general vocabulary (PPVT).

	**Story memory**			
	***Estimate***	***SE***	***t***	***p***
(Intercept)	**7.85**	0.54	14.41	**<0.001**
Group (ES)^a^	0.08	0.79	0.10	0.921
Group (IES)^a^	−0.48	0.72	−0.67	0.505
PPVT sum score^b^	**2.99**	0.31	9.71	**<0.001**
*R*^2^ (adjusted)	0.44			
*N*_subjects_	126			

*The model was fitted with the lm-function of the *lme4* package. The factor Group was dummy coded (control group = 0). The intercept represents the score of the control group at pretest. ^*a*^Reference group = control group; ^*b*^centered. Bold face indicates significant estimates (*p* < 0.05).*

### Children’s Behavior

**FIGURE 5:**
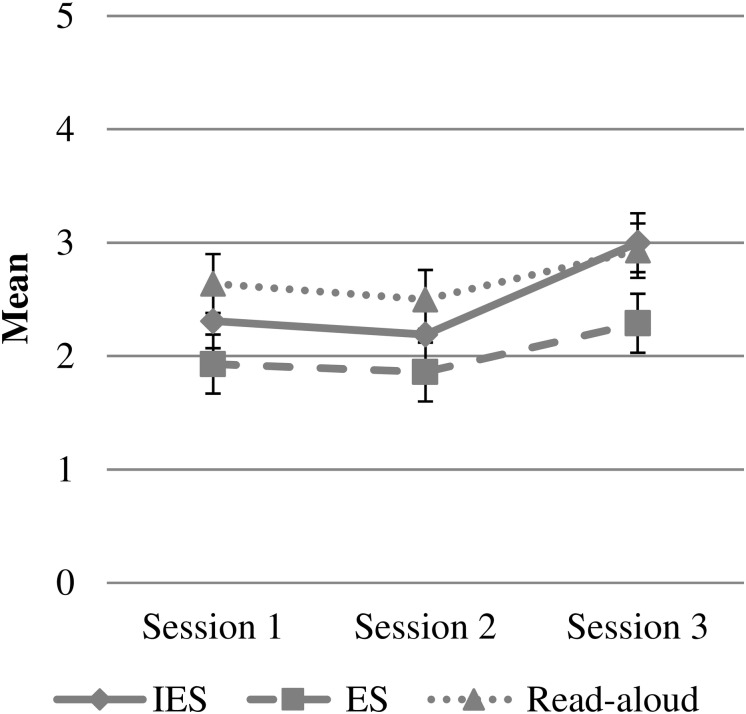
Children’s behavior (5 – *very restless*; 1 – very calm) in story sessions as a function of session and experimental group (IES = Interactive Elaborative Storytelling; ES = Elaborative Storytelling). Error bars represent ± 1 standard error.

To investigate how children behaved in the intervention groups and across the three story sessions, children’s restlessness during story sessions was analyzed exploratively with a two-way ANOVA, as a function of intervention group and story session (see Figure 5). There was a significant main effect of intervention group, *F*(2,127) = 5.23, *p* = 0.007, and story session, *F*(2,127) = 4.18, *p* = 0.017. The interaction of intervention group and story session was not significant, mean *p* = 0.90. Tukey’s *post hoc* tests showed that Elaborative Storytelling (*M* = 2.02, *SD* = 0.75) was significantly less restless than the Read-Aloud group (*M* = 2.69, *SD* = 1.07), *p* = 0.006, and marginally significantly less restless than the IES group (*M* = 2.5, *SD* = 1.09), *p* = 0.058. IES and Read-Aloud did not differ significantly, *p* = 0.626. Regarding story sessions, the third session (*M* = 2.75, *SD* = 1.18) was significantly more restless than the second session (*M* = 2.18, *SD* = 0.95), *p* = 0.020, and marginally more restless than the first session (*M* = 2.30, *SD* = 0.82), *p* = 0.078). The first and second session did not differ significantly, *p* = 0.848.

## Discussion

In the current study, we proposed a new approach to fostering vocabulary development during shared reading, called IES, and contrasted it to an Elaborative Storytelling (Vaahtoranta et al., 2018), and a Read-Aloud group in a randomized small-group study in preschool. The novel feature in this approach was to both incorporate techniques supporting word learning as well as to involve children as storytellers, with hopes that this would benefit children’s engagement in the stories and their word learning. All three experimental groups showed gains from pre- to posttest on both receptive and expressive target words. For expressive target words, the IES group scored marginally significantly higher than the control group, after controlling for general vocabulary. However, no interactions of experimental group and time were found for either receptive or expressive target vocabulary, and our hypothesis that the IES group would have the largest gains was thus not confirmed. After controlling for general vocabulary, the three groups also scored similarly on a measure of story memory. In the Elaborative Storytelling group, children were least restless during story sessions.

Our rationale in conducting the study was to foster word learning by (a) increasing activity in the shared reading situation that centers around the story by involving children as storytellers and (b) by including elaborative instructional techniques (Vaahtoranta et al., 2018). Previous literature has shown that instructional techniques (Marulis and Neuman, 2010) and active involvement of children in shared reading or other story activities can have positive effects on their language skills, especially on expressive vocabulary (Mol et al., 2008). Positive effects of retelling by children have been demonstrated for narrative skills (Nielsen and Friesen, 2012; Spencer et al., 2014; Nicolopoulou et al., 2015; Licandro, 2016) and we were interested in whether this would also lead to positive effects on expressive and receptive vocabulary. IES could thus be an alternative or supplementary approach to dialogic reading.

In testing this idea, we incorporated key design features worthy of reiterating. First, by including the Read-Aloud and the Elaborative Storytelling group, we controlled for effects of simple reading of a story on the one hand, and for effects of having facilitative techniques for word-learning on the other. This allowed us to tease out any added effect of children’s active storytelling. Second, we included measures of both receptive and expressive target word knowledge as well as story memory in order to measure not only language outcomes but also story comprehension (Walsh and Hodge, 2016). Third, we not only measured children’s language skills but also some aspects of their behavior in the story sessions, which provides information aside from cognitive learning gains on the shared-reading situations.

The finding that groups did not differ in vocabulary gains was surprising. For expressive target words, the effect is descriptively in the hypothesized direction, with IES and ES scoring higher than the read-aloud group, this, however, fails to reach significance. A possible reason is the duration of the intervention, which may have been too short to elicit group differences. It could furthermore be attributable to low variance in the data – the data were plagued with floor effects. The expressive task seemed to be too difficult for many children, which is somewhat surprising, since it has been implemented in other research (Coyne et al., 2007; Loftus et al., 2010; Lenhart et al., 2019).

A possible reason for this could lie in the difficulty of chosen target words: there were two target words for which none of the children scored any points at posttest, and some more items that had very low overall scores. We chose low-frequency words to make sure that children were not familiar with them before the experiment and to allow children to learn these during the story sessions. Although rendering it less likely that children would have previously learned the words, the downside of choosing low frequency target words is that these may be more difficult to learn, leading to floor effects A *post hoc* analysis of target word gains as function of word class showed a large effect of word class and a marginal interaction of word class and time, with nouns scoring higher and showing larger gains. Thus, effects might have differed with more concrete target words. Children scored considerably higher on the easier filler items that were included in the task to avoid frustration (e.g., *phone*, *tree*, *tired*), however, even here some children had difficulties responding to the questions and scored very low. This is probably partly attributable to language background – children who spoke an additional language at home scored considerably lower than monolinguals on filler items (*d* = 0.42). However, monolingual children partly had difficulties as well, even with the easy filler items. Such a task requires a certain level of metacognitive abilities as well as expressive language skills; possibly some children in our sample had not yet reached a certain threshold to complete this kind of task.

The missing difference between groups in the receptive target-word task is more puzzling and seems to stand, at first glance, in contrast to existing literature, which has shown that gains are higher when instructional techniques are employed (Marulis and Neuman, 2010). Receptive vocabulary tasks have, however, been found to be less sensitive at detecting intervention effects, with some studies finding larger effects for expressive tasks (Sénéchal, 1997) or no group differences in receptive tasks (Loftus et al., 2010). As stated above, the employed techniques may have not been intensive enough to lead to differences between groups on the receptive task, and the intervention would thus have to be longer to achieve this. This could also be the reason for the missing group differences in the story memory task as well.

Indeed, it is perhaps initially surprising that the IES condition did not result in greater gains in story memory. The greater activity of the children during the retelling might well have led to a better encoding of the story content. Perhaps, however, the story memory measure was not sufficiently sensitive to detect group differences and that such differences would first arise through a greater number of experimental sessions. Future work should pursue these possibilities with longer interventions.

One reason for the failure to find a difference between IES and Elaborative Storytelling is that while the children were able to train their expressive language through storytelling in interactive storytelling, this could have led to a less frequent appearance of the target words in the second and third session. The experimenters were instructed to talk about the target words also in later sessions if children, for example, mentioned synonyms of them in their storytelling, or if they told a part of the story but did not mention the word. The experimenters were instructed to for example say, “Do you remember what we called that in the story?,” and then repeat the word if children did not remember. Nevertheless, in the ES condition, target words appeared more reliably, since the stories were read aloud in every session.

Another possible reason for not finding additive effects of IES could lie in the actual amount each child could actively participate in replying to questions and telling the story. In a group of four children, each child would potentially tell 25% of the story and listen to 75% of the story; during the third session mainly told by other children. In larger groups, the individual share in telling the story would be correspondingly smaller. Although there is some evidence showing the usefulness of peer-interventions (Licandro, 2016), it is likely that children’s storytelling is considerably simpler and linguistically less rich than written stories. The reduced exposure to the original stories in IES compared to Elaborative Storytelling could thus potentially have decreased differences between the groups in word learning. It is arguably a common challenge in group settings to ensure active participation by all children, which can be strived for but not fully guaranteed. One-on-one settings guarantee the active participation of the child – these, however, often lack feasibility in preschool settings.

We were also interested in children’s behavior during the story sessions and whether it varied between intervention groups. An exploratory investigation of children’s restlessness during story sessions showed that children were least restless in the Elaborative Storytelling condition and that the third session was the most restless. The difference between Elaborative Storytelling and the read-aloud group could be a consequence of the techniques employed in the Elaborative Storytelling condition but not in read-aloud that were designed to foster word learning but also keep children engaged in the story. Because of the simple repeated reading, the Read-Aloud group might have lost interest in the story, which in turn could have led to more disruptive behavior. An alternative explanation for the higher restlessness in the Read-Aloud group is the composition of the groups. Despite random assignment, the read-aloud group displayed lower general vocabulary than the other groups. Furthermore, although only marginally significant, mothers of the children in the read-aloud group had the lowest education of the three groups. Thus, children could have been overstrained and hence lost interest in the story session. Alternatively, due to the lower maternal education, they might not have been as much used to shared reading as children from highly educated homes (Van Steensel, 2006). When observing shared-reading situations in low-SES vs. high-SES preschools, the difference in the ability to concentrate on the stories and thus the amount of disruptive behavior is painfully obvious. Shared reading could be a helpful tool for these children to learn to focus and to use their imagination. But perhaps especially these children need additional prompts or interactivity to keep them engaged in the story.

The IES group did not differ significantly from the read-aloud group when it comes to children’s restlessness and was descriptively between ES and the read-aloud group. This could be a result of including children as storytellers, which invites more overall restlessness into the story session – managing the storytelling of a group of children can be challenging. However, a problem with this measure of children’s behavior is that it does not discriminate between restlessness due to children’s boredom or inattentiveness, and restlessness due to the nature of the intervention group. We attempted to measure children’s interest in the story session as well, which proved to be difficult to rate, reflected in the low inter-rater reliability. Thus, this could unfortunately not be included in the analysis. The fact that the third session was more restless in all intervention groups suggests that children were not as engaged in the activity in the third session, which corresponds with reports of students conducting the story sessions. This finding should be taken into consideration in future research, namely, that reading the same stories on three consecutive days could lead to boredom effects.

### Limitations and Implications for Future Research

A problem we encountered was that despite random assignment, children in the read-aloud group displayed a lower receptive vocabulary than the other experimental groups. Moreover, the selection of difficult target words could have restricted effects of the story sessions and children may have needed more time and support to learn them. A further problem was the low inter-rater reliability in measuring children’s engagement, or interest, in the story sessions. This was an attempt to disentangle children’s restlessness from their engagement because some children are restless although they are engaged/interested in the activity. However, this proved to be a difficult task in the current study and was in contrast to Vaahtoranta et al. (2018), where this measure reached sufficient inter-rater reliability. Future studies should use videotapes of the shared-reading sessions so that children’s behavior can be rated with more reliability.

It is possible that interactive storytelling was an unfamiliar and challenging situation to the children. This could have had two consequences: on the one hand, this might have taken the attention too much away from target words, and on the other hand, children’s cognitive capacity was possibly exhausted by the storytelling. It is conceivable that positive effects would be measurable in a longer intervention where children would have the chance to get used to this form of shared reading. A longer investigation would also be warranted in order to investigate not only short-term vocabulary gains but also long-term and possible transfer effects. A short-term study such as the current study is not expected to have effects on narrative skills – a longer intervention including storytelling by children, however, could. An investigation of narrative skills as result of a long-term IES intervention is thus warranted.

However, the current study provides differential findings that may inform the practice of language interventions. Story exposure, regardless of whether read-aloud, or accompanied with elaborative and active storytelling on the part of the children, improves vocabulary development. The kind of technique employed is also likely to have an effect on child behavior, with elaborative techniques appearing to engage children more heavily in the story itself. Future work should clarify the long-term effects of both story delivery and more detailed analyses of child behavior. Perhaps considering the entire picture will open new avenues in optimizing language interventions.

## Data Availability

The datasets generated for this study are available on request to the corresponding author.

## Ethics Statement

The protocol was approved by the ethics committee of the Institute for Psychology of the Philosophical Faculty II at the Julius-Maximilians-University of Würzburg. Written informed consent was obtained from the parents of all participants.

## Author Contributions

All authors were involved in the designing of the study. EV and JL carried out the experiment and prepared the data. EV analyzed the data and wrote the manuscript with input from all other authors.

## Conflict of Interest Statement

The authors declare that the research was conducted in the absence of any commercial or financial relationships that could be construed as a potential conflict of interest.
